# Spontaneous cleavage of proteins at serine and threonine is facilitated by zinc

**DOI:** 10.1111/acel.12428

**Published:** 2016-01-11

**Authors:** Brian Lyons, Ann H. Kwan, Roger J.W. Truscott

**Affiliations:** ^1^ Illawarra Health and Medical Research InstituteUniversity of WollongongNorthfields AveWollongongNSW2522Australia; ^2^ Save Sight InstituteUniversity of SydneySydney Eye Hospital8 Macquarie StSydneyNSW2000Australia; ^3^ School of Molecular BioscienceUniversity of SydneySydneyNSW2006Australia

**Keywords:** ageing, aging, Alzheimer's disease, lifespan, longevity, neurodegenerative disease, protein chemistry, proteomics

## Abstract

Old proteins are widely distributed in the body. Over time, they deteriorate and many spontaneous reactions, for example isomerisation of Asp and Asn, can be replicated by incubation of peptides under physiological conditions. One of the signatures of long‐lived proteins that has proven to be difficult to replicate *in vitro* is cleavage on the N‐terminal side of Ser residues, and this is important since cleavage at Ser, and also Thr, has been observed in a number of human proteins. In this study, the autolysis of Ser‐ and Thr‐containing peptides was investigated with particular reference to discovering factors that promote cleavage adjacent to Ser/Thr at neutral pH. It was found that zinc catalyses cleavage of the peptide bond on the N‐terminal side of Ser residues and further that this process is markedly accelerated if a His residue is adjacent to the Ser. NMR analysis indicated that the imidazole group co‐ordinates zinc and that once zinc is co‐ordinated, it can polarize the carbonyl group of the peptide bond in a manner analogous to that observed in the active site of the metalloexopeptidase, carboxypeptidase A. The hydroxyl side chain of Ser/Thr is then able to cleave the adjacent peptide bond. These observations enable an understanding of the origin of common truncations observed in long‐lived proteins, for example truncation on the N‐terminal side of Ser 8 in Abeta, Ser 19 in alpha B crystallin and Ser 66 in alpha A crystallin. The presence of zinc may therefore significantly affect the long‐term stability of cellular proteins.

## Introduction

Long‐lived proteins such as those found in the brain, heart, connective tissue, lung and the lens are subject to many age‐related modifications including racemization (Shapira *et al*., 1988; Hooi & Truscott, 2011), crosslinking (Wang *et al*., 2014) and isomerization of Asp and Asn (Shapira *et al*., 1988; Stephenson & Clarke, 1989). However, the mechanisms that underpin some of these processes are still poorly understood. One such modification, which is commonly observed in long‐lived proteins, is truncation at the N‐terminal side of Ser and Thr residues (Lampi *et al*., 1998; Friedrich *et al*., 2012, 2000; Sergeant *et al*., 2003; Santhoshkumar *et al*., 2008; Vlad *et al*., 2011; Su *et al*., 2012). In some tissues, this process could be enzymically mediated; however, proteases with specificity for cleavage next to Ser/Thr are rare. One example is a sterol‐regulated protease present in the endoplasmic reticulum (Duncan *et al*., 1997). In the adult human lens, no active proteases remain in the centre (Zhu *et al*., 2010) due to the particular growth pattern of the lens, so this is an excellent tissue to examine protein cleavage processes that occur spontaneously. In this tissue, several cleavage events on the N‐terminal side of Ser and Thr have been documented (Lampi *et al*., 1998; Schey *et al*., 2000; Santhoshkumar *et al*., 2008; Su *et al*., 2012).

Previous mechanistic studies (Lyons *et al*., 2011, 2014) involved the use of elevated temperatures, extended incubation periods and/or nonphysiological pH to induce truncation at Ser in model peptides. This study demonstrates for the first time a mechanism by which this process can readily occur under physiological conditions in the presence of zinc. Zinc is a major trace metal in biological systems and is known to play a role in many physiological processes (Laity *et al*., 2001) (Vallee & Auld, 1990) including cell signalling (Hajo & Wolfgang, 2010). It is an abundant metal in the brain with concentrations estimated at between 0.09 mM and 0.30 mM zinc (Hu & Friede 1968) and is also present in the lens at 0.13 mm (Rasi *et al*., 1992). Zinc has been implicated in some protein misfolding diseases (Rakhit & Chakrabartty, 2006; Malgieri & Grasso, 2014). This study indicates that although zinc is necessary for normal biological function, it can adversely affect the stability of long‐lived proteins in the cell by promoting spontaneous cleavage.

## Results

Cleavage of long‐lived proteins on the N‐terminal side of Ser has been reported in proteins from a number of tissues (Table 1) (Smith *et al*., 1992; Takemoto, 1995; Lampi *et al*., 1998; Santhoshkumar *et al*., 2008; Su *et al*., 2010, 2012; Friedrich *et al*., 2012) although this process has been most well documented in the lens. In the human lens, an abundant peptide is derived from the age‐related truncation of alpha B crystallin between residues 18 (His) and 19(Ser) (Santhoshkumar *et al*., 2008). To see whether this cleavage could be replicated *in vitro,* a peptide containing this sequence (^16^PFHSPS^21^) was incubated and the products characterized by mass spectrometry and NMR spectroscopy. In this and subsequent investigations, 37°C was used to mirror physiological temperature and 60°C was used to accelerate reaction rates.

**Table 1:** Long‐lived proteins known to truncate at the N‐terminus of Ser/Thr

Protein	Truncates at	References
Aβ	Ser 8	Sergeant *et al*. (2003)
α synuclein	Thr 72	Vlad *et al*. (2011)
αA crystallin	Thr 13	Su *et al*. (2012), Smith *et al*. (1992), Su *et al*. (2010)
αA crystallin	Ser 20	Su *et al*. (2012)
αA crystallin	Ser 42	Su *et al*. (2012)
αA crystallin	Thr 43	Su *et al*. (2010)
αA crystallin	Ser 51	Su *et al*. (2012)
αA crystallin	Thr 55	Smith *et al*. (1992)
αA crystallin	Ser 66	Su *et al*. (2012), Santhoshkumar *et al*. (2008), Su *et al*. (2010)
αA crystallin	Ser 81	Su *et al*. (2012), Santhoshkumar *et al*. (2008), Su *et al*. (2010)
αA crystallin	Thr 148	Su *et al*. (2012)
αA crystallin	Ser 169	Takemoto (1995)
αA crystallin	Ser 173	Su *et al*. (2012), Takemoto (1995)
αB crystallin	Ser 19	Su *et al*. (2012), Santhoshkumar *et al*. (2008)
αB crystallin	Ser 21	Su *et al*. (2012)
αB crystallin	Ser 35	Santhoshkumar *et al*. (2008)
αB crystallin	Ser 43	Su *et al*. (2012)
αB crystallin	Ser 45	Su *et al*. (2012), Santhoshkumar *et al*. (2008), Su *et al*. (2010)
αB crystallin	Ser 66	Su *et al*. (2012), Santhoshkumar *et al*. (2008)
αB crystallin	Thr 134	Su *et al*. (2012)
αB crystallin	Ser 153	Su *et al*. (2012)
αB crystallin	Thr 158	Smith *et al*. (1992)
β A3 crystallin	Ser 27	Su *et al*. (2012)
β A3 crystallin	Ser 59	Santhoshkumar *et al*. (2008)
β B1 crystallin	Ser 2	Lampi *et al*. (1998), Su *et al*. (2012)
β B1 crystallin	Thr 38	Su *et al*. (2012)
β B1 crystallin	Ser 185	Su *et al*. (2012)
β B2 crystallin	Ser 3	Lampi *et al*. (1998)
β B2 crystallin	Ser 14	Su *et al*. (2012)
β B2 crystallin	Ser 31	Su *et al*. (2012)
β B2 crystallin	Thr 91	Su *et al*. (2012)
β B2 crystallin	Ser 148	Su *et al*. (2012)
β B2 Crystallin	Ser 186	Su *et al*. (2012)
γS crystallin	Ser 2	Su *et al*. (2012)
γS crystallin	Ser 167	Santhoshkumar *et al*. (2008), Friedrich *et al*. (2012)
γS crystallin	Ser 172	Friedrich *et al*. (2012)
AQP0	Ser 6	Schey *et al*. (2000)
AQP0	Ser 245	Schey *et al*. (2000)
AQP0	Thr 252	Schey *et al*. (2000)
IgG1	Ser 219	Cohen *et al*. (2007)

**Figure 1:**
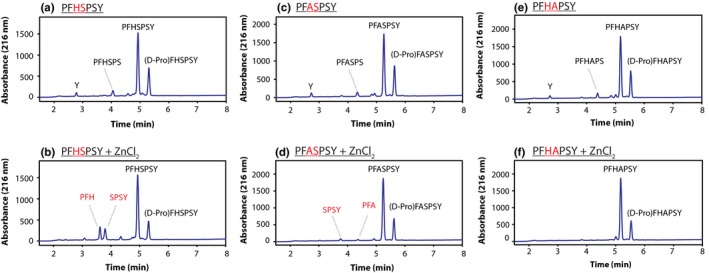
Zinc‐induced truncation at Ser and the effect of amino acid sequence. One representative HPLC trace from each triplicate is shown following the incubation of PFHSPSY (a,b), PFASPSY (c,d) and PFHAPSY (e,f) at 60°C for 14 days. Racemization of N‐terminal residues following incubation (i.e. D‐ProFHASPY) has been characterized in a previous publication (22). Peptides were incubated in 50 mm Tris, pH 7.4 ± 2 mm ZnCl_2_ as indicated. Of interest, cleavage of the C‐terminal Tyr residue was more evident in samples that lacked Zn. Detection at 216 nm.

**Figure 2:**
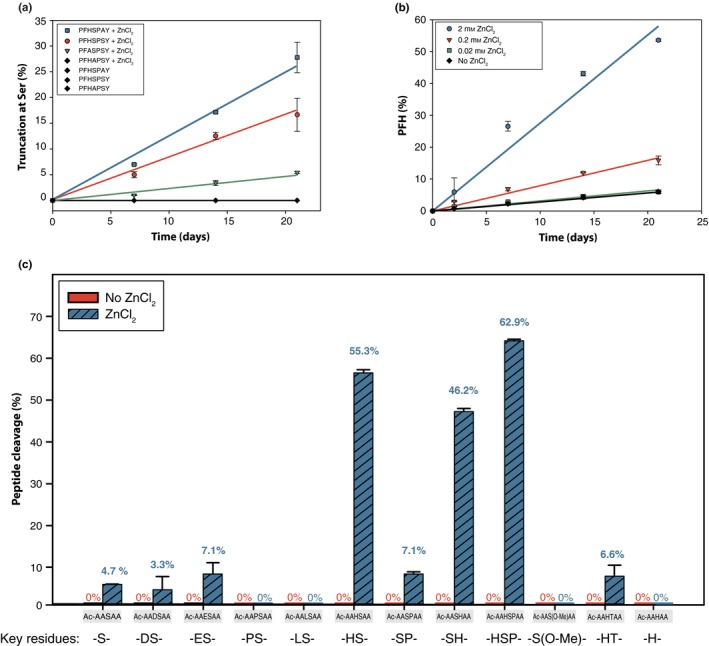
Time course illustrating the effect of (a) peptide sequence on cleavage and (b) zinc concentration on PFHSPAY cleavage next to Ser. (c) Bar graphs highlighting the effect of sequence following incubation of 12 peptides (Ac‐AASAA, Ac‐AADSAA, Ac‐AAESAA, Ac‐AAPSAA, Ac‐AALSAA, Ac‐AAHSAA, Ac‐AASPAA, Ac‐AASHAA, Ac‐AAHSPAA, Ac‐AA(O‐Me)AA, Ac‐AAHTAA and Ac‐AAHAA) for 21 days. All peptides were incubated in 50 mm Tris, pH 7.4 ± 2 mm ZnCl_2_ at 60°C. The position of His was statistically significant with respect to the rate of cleavage: HS vs. SH (*P *= 0.000096), as was the presence of C‐terminal Pro: HS vs. HSP (*P *= 0.000090), SP vs. HSP (0.000000014).

As dipeptide cleavage had been reported to be catalysed by divalent metals, in particular zinc (Yashiro *et al*., 2003), zinc was incorporated in the current study. As shown in Fig. 1a,b, the addition of zinc resulted in a specific cleavage of the N‐terminal peptide bond at Ser 4, which was not observed in the control incubation which lacked zinc. To investigate the role of adjacent residues, additional peptides (PFASPSY, PFHAPSY and PFHSPAY) were incubated. Replacing the His residue with an Ala resulted in significantly less truncation at Ser 4 (Fig. 1c,d). To confirm the involvement of the side chain hydroxyl group, replacement of Ser 4 with Ala resulted in complete loss of truncation despite the presence of a His (Fig. 1e,f). Interestingly, although products derived from scission at Ser 4 were abundant, little cleavage of the peptide at Ser 6, which is spatially separate from the His residue, was detected. A time course showing the amount of truncation, which occurred in each incubation, is shown in Fig. 2a.

To further investigate the role of the Ser hydroxyl group in this cleavage, Ac‐YASP and a variant where the hydroxyl group was methylated, Ac‐YAS(O‐Me)P, were incubated in the presence and absence of zinc. These tetra peptides were chosen in order to reduce unrelated reactions that can occur following incubation at high temperatures and an alpha N‐acetyl group was also added in order to prevent modifications such as racemization / diketopiperazine formation (Goolcharran & Borchardt, 1998; Lyons *et al*., 2013). While some truncation at Ser was observed for Ac‐YASP (1.6 pmoles h^−1^), the rate increased significantly with the presence of zinc (9 pmoles h^−1^), following incubation at 60°C; however, no truncation occurred with Ac‐YAS(O‐Me)P even when incubated with zinc (data not shown).

The effect of zinc concentration was examined by incubating PFHSPAY in 2 mm, 0.2 mm and 0.02 mm ZnCl_2._ (Fig. 2b) These incubations were performed in water (adjusted to pH 7.4) in order to eliminate any possible effect of Tris binding to the zinc (Pena *et al*., 1990) and influencing the reaction. At 2 mm ZnCl_2_, the initial rate of Ser truncation was 200.4 pmoles h^−1^ and this dropped significantly as the amount of zinc was reduced to 0.2 mm (60.9 pmoles h^−1^) and 0.02 mm (23.4 pmoles h^−1^). A small amount of truncation was also observed for the zinc‐free incubation (19.5 pmoles h^−1^). Clearly, the rate of truncation at Ser is dependent on the concentration of zinc and cleavage occurred in the absence of Tris. When Tris was replaced by 50 mm MOPS, cleavage of PFHSPAY still took place although the rate was reduced by approximately 18%. MOPS is a goods buffer and is noted for not forming metal complexes (Yu *et al*., 1997).

To study the effect of neighbouring residues on the degree of cleavage, 12 peptides (Ac‐AASAA, Ac‐AADSAA, Ac‐AAESAA, Ac‐AAPSAA, Ac‐AALSAA, Ac‐AAHSAA, Ac‐AASPAA, Ac‐AASHAA, Ac‐AAHSPAA, Ac‐AA(O‐Me)AA, Ac‐AAHTAA and Ac‐AAHAA) were incubated in the presence and absence of zinc. A bar graph illustrating the amount of truncation at the N‐terminal side of Ser following 21‐day incubation at 60°C ±ZnCl_2_ is shown in Fig. 2c. The inclusion of a His residue on either side of the Ser resulted in a significant increase in the amount of truncation at Ser and the inclusion of a Pro adjacent to the His increased the degree even further. The relatively small amount of zinc‐induced truncation observed for Ac‐AASAA suggests that some truncation can occur in some circumstances even in the absence of an adjacent His residue.

Although His exerted the strongest effect, adjacent Asp or Glu residues, both with a known ability to co‐ordinate to zinc (Vallee & Auld, 1993), also induced cleavage on the N‐terminal side of Ser. A computational study of zinc (II) interactions (Trzaskowski *et al*., 2008) had predicted a high binding affinity for Pro residues, but if this occurs, cleavage was not observed experimentally under our conditions. Insertion of a Pro residue resulted in less scission next to Ser when His was not present. In line with previous work at high pH (Lyons *et al*., 2014), Ser‐Pro was more susceptible than Pro‐Ser. Interestingly, when Ser was replaced with Thr, truncation was also observed, although to a reduced extent. This is in line with the involvement of the side chain hydroxyl group in the cleavage mechanism.

**Figure 3:**
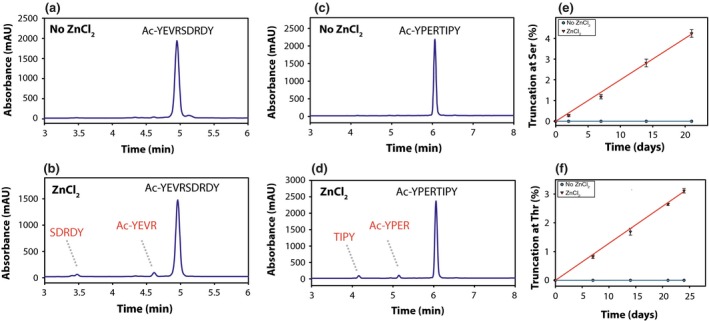
Truncation at Ser/Thr residues of peptides. HPLC traces of (a) Ac‐YEVRSDRDY, (b) Ac‐YEVRSDRDY + zinc, (c) Ac‐YPERTIPY and (d) Ac‐YPERTIPY + zinc following 21‐day incubation at 60°C. The rate of cleavage beside Thr (f) in this peptide was slower, but comparable with that next to Ser (e). Peptides were incubated in 50 mM tris pH 7.4, +/− 2 mM ZnCl_2_.

In Fig. 2c, one peptide containing Thr was also shown to also cleave in the presence of zinc. To examine cleavage at Thr in more detail, Ac‐YPERTIPY was employed. Ac‐YPERTIPY incorporates a sequence region of alpha B crystallin (154–160) where age‐related truncation is known to occur at Thr 158 (Smith *et al*., 1992). Its cleavage was compared with that of a Ser‐containing peptide derived from alpha A crystallin, Ac‐YEVRSDRDY. Ac‐YEVRSDRDY incorporates a sequence of alpha A crystallin (63–69) which is known to truncate at Ser 66 with age (Santhoshkumar *et al*., 2008; Su *et al*., 2012). As shown in Fig. 3, for both peptides, truncation was observed selectively on the N‐terminal sides of either Ser or Thr and only if zinc was present. The rates of both the Ser and Thr cleavages were comparable.

**Figure 4:**
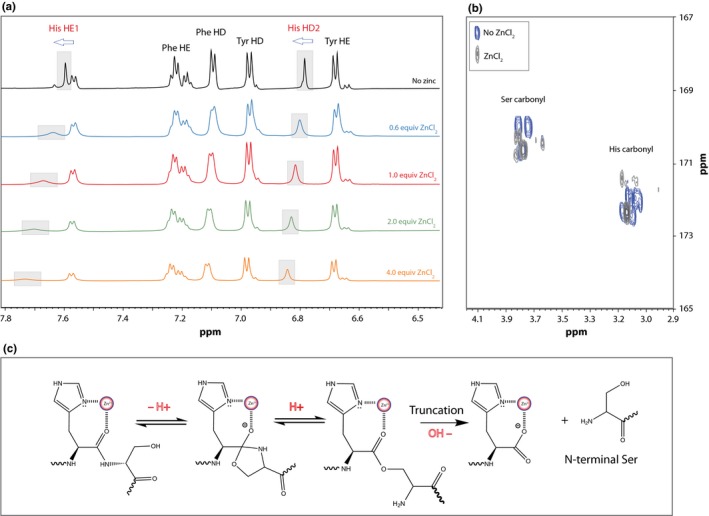
NMR titration experiment showing the effect of zinc addition on (a) the aromatic proton region of PFHSPSY and (b) the Ser and His carbonyl regions of PFHSPSY. PFHSPSY was dissolved (2 mg mL^−1^) in 20 mm deuterated Tris pH 7.4 (in D_2_O) and ZnCl_2_ (in 20 mm deutrated Tris pH 7.4 / D_2_O) was added stepwise from 0.6 to 4 molar equivalents. (c) A mechanism that accounts for zinc‐induced truncation at the N‐terminal side of Ser residues. An analogous mechanism is proposed for Thr.

NMR spectroscopy was then used to investigate the precise mechanism of zinc binding. PFHSPSY was dissolved in 20 mm deuterated Tris pH 7.4 in D_2_O and TOCSY and HSQC spectra were recorded and used to assign all the peaks in the peptide (data not shown). ZnCl_2_ (in 20 mm Tris pH 7.4 in D_2_O) was then titrated in from 0.6 to 4 molar equivalents, and proton spectra were recorded. As seen in Fig. 4a, significant deshielding of His HE1 and HD2 protons was observed which implies a strong coordination through the N1 imidazole nitrogen, while the peaks from other residues were unaffected. These results are consistent with the findings of Nair *et al*. (2009) who studied the interactions of zinc and His using NMR spectroscopy. A 2D HMBC spectrum revealed that the carbonyl groups corresponding to His and Ser residues were most affected following addition of zinc (Fig. 4b) which further supports an interaction within this region of the peptide. A mechanism that illustrates the coordination of zinc by His and its role in peptide bond cleavage by the hydroxyl side chain of Ser is shown in Fig. 4c.

**Figure 5:**
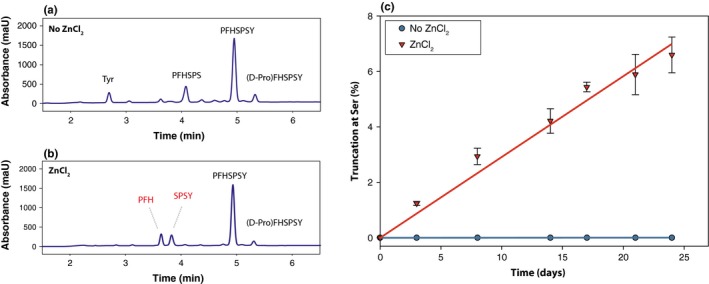
Representative HPLC traces following incubation of (a) PFHSPSY and (b) PFHSPSY + ZnCl2 at 37°C for 21 days. (c) Time course illustrating the effect of zinc on truncation at Ser 4. PFHSPSY was incubated in 50 mm Tris, pH 7.4 ± 2 mm ZnCl_2_ as indicated.

**Figure 6:**
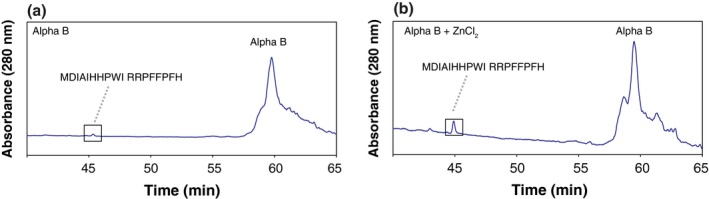
Truncation of recombinant alpha B crystallin at Ser is induced by zinc. (a) Incubation of recombinant (a) alpha B crystallin and (b) alpha B crystallin + zinc at 60°C for 14 days. Alpha B was incubated in 50 mm Tris, pH 7.4 ± 2 mm ZnCl_2_ as indicated.

To demonstrate that the reactions characterized here can also occur at biologically‐relevant temperatures, PFHSPSY, which incorporates the sequence of alpha B crystallin 16–21, was incubated at 37°C and pH 7.4 (Fig. 5a,b) and the rate of truncation was plotted. After 7‐day incubation, ~5% of PFHPSY had been truncated at Ser (Fig. 5c) in the presence of zinc. To show that the effect of zinc on peptides also applies to intact proteins, recombinant alpha B crystallin was incubated in the presence and absence of zinc and the incubations analysed by HPLC. As can be seen in Fig. 6, the incubation that incorporated zinc contained a prominent peptide peak that was much reduced in the zinc‐free incubation. Analysis of this by tandem mass spectrometry revealed it to have the sequence MDIAIHHPWI RRPFFPFH. This corresponds to the major cleavage site of alpha B crystallin from aged human lenses (Su *et al*., 2010, 2012) and is also the Ser truncation site investigated using the model peptide PFHSPSY (Fig. 1a).

## Discussion

This study describes a novel mechanism by which truncation at the N‐terminal side of Ser and Thr residues in peptides and proteins is mediated via zinc. Cleavage next to these residues has been often observed in long‐lived proteins (Smith *et al*., 1992; Takemoto, 1995; Lampi *et al*., 1998; Santhoshkumar *et al*., 2008; Su *et al*., 2010, 2012; Friedrich *et al*., 2012). Our work extends a publication on dipeptides by Yahiro *et al*. (Yashiro *et al*., 2003) where they observed cleavage adjacent to Ser was assisted by a number of divalent metal ions, in particular, zinc. Their proposed mechanism involved coordination of the metals to the free alpha amino group of the dipeptide, as well as to the carbonyl group of the peptide bond on the N‐terminal side of the Ser residue. Peptide bond hydrolysis was mediated by the hydroxyl group of the Ser side chain.

In the current study, we show that an analogous reaction can take place at internal Ser and Thr residues in peptides and proteins. In these cases, co‐ordination to an alpha amino group is not possible, but other amino acid chelators in particular the side chain of His can act in a comparable manner. Based on the dipeptide data (Yashiro *et al*., 2003), it is very likely that other divalent metals such as Ni, Ca, Cd and Mg can also accelerate autolysis adjacent to Ser residues in long‐lived proteins; however, this was not investigated in this study. To characterize the mechanism of cleavage, a number of factors were considered.

A His residue on either side of Ser significantly increased the rate of truncation (Fig. 2). The effect of other adjacent residues in this study was less apparent, thus highlighting the key role played by His. The presence of a Pro residue in addition to a His and Ser resulted in the highest level of truncation.

Replacement of the Ser residue with an Ala abolished truncation even when an adjacent His residue was present.. When the Ser hydroxyl group was replaced by a methyl ether, there was complete loss of truncation, even in the presence of zinc. Similar results were obtained in MOPS buffer, thus suggesting that this cleavage is not buffer dependant.

NMR spectroscopy enabled a detailed study of the zinc–peptide interaction. As observed in Fig. 4, the significant shift and relative change in intensity of His‐HE1 and His‐HE2 protons strongly suggested His as the major binding site and these findings are consistent with Nair *et al*. (Nair *et al*., 2009). It is proposed that once zinc is co‐ordinated to the peptide by His that the zinc is then in a position to polarize the carbonyl group of the peptide bond in a manner analogous to that observed with carboxypeptidase A (Barber & Fisher, 1972) This polarization enables cleavage of that peptide bond by the hydroxyl group of Ser or Thr.

To support the physiological relevance of this modification, a peptide sequence analogous to the region of human alpha B crystallin, known to truncate N‐terminally at Ser (^16^PFH‐SPS^21^), was shown to truncate at Ser in the presence of zinc at 37°C (Fig. 5). The ability of intact proteins to undergo this same cleavage was demonstrated by incubating alpha B crystallin and showing that truncation at Ser 18 occurred in the presence of zinc (Fig. 6).

While proteins such as elastin in heart and lung, collagen in skeletal and connective tissues and lens crystallins have been recognized as being long‐lived, or lifelong, for some time (see e.g. Cloos & Christgau (2002); Truscott (2010) for reviews, the list of long‐lived proteins is expanding (Toyama *et al*., 2013; )). The findings from the current study have implications for every cell and tissue that contains long‐lived proteins. All cells, as well as extracellular fluids, contain zinc, so our data indicate that under these conditions, susceptible Ser and Thr residues in polypeptides can be prone to nonenzymatic cleavage. The likelihood of such scission is dependent on adjacent residues, in particular His, and may well be influenced by protein conformation. Such spontaneous proteolysis may well have consequences for the function of biological tissues and is likely to increase in postmitotic cells as a function of age.

## Materials and methods

### Peptides

All peptides were synthesized by GLS Biochem (Shanghai, China). TFA (Sigma, St Louis, MO, USA) was spectrophotometric grade. All solutions were prepared in Milli‐Q water (Waters, Billerica, MA, USA).

### Peptide incubations

Peptides were dissolved in triplicate (1 mg mL^−1^ pH 7.4) in 100 mm phosphate buffer, 50 mm Tris or 50 mm MOPS buffer at 37°C or 60°C as specified in the text. 37°C was used to mirror physiological temperature and 60°C was used to accelerate reaction rates. For the 37°C incubations, a drop of chloroform was added to prevent microbial growth. ZnCl_2_ was added to some incubations (as described in the text). Aliquots (20 μL) were taken at regular time points and analysed by HPLC.

### Identification of peptides

Unknown peaks which formed in each incubation were isolated using HPLC and characterized using ESI and MALDI mass spectrometry. Characterization was further supported by synthesizing peptide standards of each proposed cleavage product and checking that their HPLC retention times and mass spectra matched.

### Quantification of peptides

For peptides which contained a Tyr residue, the amount of cleavage was calculated by comparing the peak area of the modified peptide (280 nm) at a given time point with the peak area of the original peptide at T = 0. For other peptides, standard curves were generated by injecting increasing amounts of a synthetic peptide standards and calculating the peak area at either 216 nm or 257 nm.

### Alpha B crystallin incubations

Recombinant human alpha B crystallin (1 mg mL^−1^) was incubated at 60°C ± 2 mm ZnCl_2_ in 50 mm Tris pH 7.4 containing 1M guanidine HCl. Aliquots (50 μL) were analysed by HPLC.

### HPLC analysis and quantification

An Agilent 1100 HPLC system (Agilent Technologies, Santa Clara, CA, USA) controlled using ChemStation software and equipped with a PDA detector was used. Incubations were monitored at 280 and 216 nm. Separation of the peptides was achieved using a Jupiter Proteo 4 μm 90 Å column (150 mm × 4.6 mm ID) at 40°C. The gradient was 0% B (0.1% TFA in acetonitrile) to 60% B (0.1% TFA in acetonitrile) over 15 min with a flow rate of 2 mL min^−1^. The degree of modification was calculated based on the moles of each peptide formed as a percentage of moles of peptide present at the start of the incubation. The error bars refer to the standard deviation of three replicates. Separation of peptides in the alpha B crystallin incubations was achieved using the same gradient but run over two hours.

### Semi‐preparative HPLC purification

A Shimadzu Prominence HPLC system (Shimadzu, Kyoto, Japan) controlled by Shimadzu Class VP software equipped with a UV‐vis detector (SPD‐20A) was used. Purification of the peptides was achieved using a Phenomenex Kinetex (Torrance, CA, USA; 100 mm × 4.6 mm ID) 2.6 μm 100 Å column at ambient temperature and was monitored at 280 and 216 nm. The gradient was 0% B (0.1% TFA in acetonitrile) to 60% B (0.1% TFA in acetonitrile) over 110 min.

### Analysis by MALDI‐MS/MS mass spectrometry

MALDI‐MS analysis was performed using a Shimadzu (Nakagyo‐ku, Kyoto, Japan) Axima TOF2 mass spectrometer used in reflectron positive ion mode. Peptides were prepared in α‐cyano‐4‐hydroxycinnamic acid (8 mg mL^−1^) in 80% (v/v) acetonitrile, 0.1% (v/v) TFA.

### NMR analysis

Samples were prepared in 50 mm phosphate buffer, pH 7.4, containing 1 mm 4,4‐dimethyl‐4‐silapentane‐1‐sulphonic acid and 10% D_2_O. Spectra were acquired at 25°C on an Avance III 600 MHz NMR spectrometer (Bruker, Karlsruhe, Germany) equipped with a triple‐resonance TCI cryoprobe. All NMR experiments were acquired using standard Bruker pulse sequences.

## Funding

Funding for this work was provided by NHMRC # 1008667.

## Conflict of interest

The authors do not have any conflict of interests to declare.

## Author contributions

BL, AK and RT planned the experiments; BL and AK performed the experiments; BL, AK and RT analysed the data; and BL, AK and RT wrote the manuscript.
